# Association of *HTRA1* and *ARMS2* gene polymorphisms with response to intravitreal ranibizumab among neovascular age-related macular degenerative subjects

**DOI:** 10.1186/s40246-019-0197-3

**Published:** 2019-02-22

**Authors:** Nur Afiqah Mohamad, Vasudevan Ramachandran, Hazlita Mohd Isa, Yoke Mun Chan, Nor Fariza Ngah, Siew Mooi Ching, Fan Kee Hoo, Wan Aliaa Wan Sulaiman, Liyana Najwa Inche Mat, Mohd Hazmi Mohamed

**Affiliations:** 1 Malaysian Research Institute on Ageing, Universiti Putra Malaysia, 43400 Serdang, Selangor DE Malaysia; 2 Department of Ophthalmology, Universiti Kebangsaan Malaysia Medical Centre, 56000 Cheras, Kuala Lumpur Malaysia; 3 Department of Nutrition and Dietetics, Faculty of Medicine and Health Sciences, Universiti Putra Malaysia, 43400 Serdang, Selangor DE Malaysia; 4 Department of Ophthalmology, Hospital Selayang, Lebuhraya Selayang-Kepong, 68100 Batu Caves, Malaysia; 5 Department of Family Medicine, Faculty of Medicine and Health Sciences, Universiti Putra Malaysia, 43400 Serdang, Selangor DE Malaysia; 6 Department of Medicine, Faculty of Medicine and Health Sciences, Universiti Putra Malaysia, 43400 Serdang, Selangor DE Malaysia; 7 Department of Surgery, Faculty of Medicine and Health Sciences, Universiti Putra Malaysia, 43400 Serdang, Selangor DE Malaysia

**Keywords:** Age-related macular degeneration, Age-related maculopathy susceptibility 2, High temperature requirement a serine peptidase 1, Polymorphism, Ranibizumab

## Abstract

**Background:**

The association of *HTRA1* rs11200638 and *ARMS2* rs10490924 gene polymorphisms with response to intravitreal ranibizumab therapy among neovascular AMD (nAMD) subjects in Malaysia was determined in this study, followed by the expression of *HTRA1* and *ARMS2* genes.

**Results:**

Both single nucleotide polymorphisms (SNPs) recorded a significant association between nAMD and controls with *HTRA1* rs11200638 at *P* = 0.018 (OR = 1.52, 95% CI = 1.07–215) and *ARMS2* rs10490924 at *P* < 0.001 (OR = 2.44, 95% CI = 1.75–3.42). An association was also observed in response to ranibizumab for both SNPs in a logistic regression analysis (*P* < 0.001). The mRNA levels in the *HTRA1* variant between responder and non-responder groups were significantly different for the homozygous non-risk GG genotype (*P* = 0.032).

**Conclusions:**

The *HTRA1* rs11200638 and *ARMS2* rs10490924 gene polymorphisms are associated with nAMD among Malaysians. Both gene polymorphisms were also correlated with response to intravitreal ranibizumab therapy based on visual and anatomical outcomes especially the *HTRA1* rs11200638 variant.

## Background

Age-related macular degeneration (AMD) is a complex multifactorial disease and a common cause of visual impairment among elderly populations worldwide [1]. The neovascular (or exudative) form of AMD (nAMD) is an advanced form of AMD and responsible for the majority of irreversible vision loss if left untreated. The aetiology of nAMD is multifactorial; both environmental and genetic predispositions are suggested to be involved in nAMD pathogenesis. Advanced age and smoking are the strongest modifiable risk factors reported in association with nAMD among Europeans [2, 3] and in Asian populations [4, 5]. In addition to environmental factors, genetics plays a role in the aetiology of nAMD. *Age-related maculopathy susceptibility 2* (*ARMS2* or *LOC387715*) and *high-temperature requirement A serine peptidase 1* (*HTRA1*) genes are among the susceptible genes that are well studied in most populations with contradictory findings [6–8].

The *ARMS2* and *HTRA1* genes are located nearby on the 10q26 chromosome in a strong linkage disequilibrium which could be the main reason for these inconsistent results as most genetic association studies have insufficient data on distinguishing these two genes with nAMD. The rs11200638 polymorphism at the promoter region of *HTRA1* was suggested to be related to overexpression of the gene among nAMD patients with the minor A allele, which was related to a significantly lower expression of *HTRA1* mRNA compared with the major G allele [9, 10]. However, Wang et al. showed that there are no significant changes to the *HTRA1* rs11200638 and *ARMS2* rs10490924 mRNA levels among AMD [11].

*HTRA1* encodes a heat shock serine protease and regulates the transforming growth factor-β (TGF-β) which is involved in regulating angiogenesis and extracellular matrix deposition; thus, the overexpression of *HTRA1* among nAMD was suggested to be due to the inhibition of TGF-β [12]. The *ARMS2* gene was shown to encode a function in the mitochondrial outer membrane of the retina; therefore, the rs10490924 polymorphism in the exon 1 region of the *ARMS2* gene, which causes a change of alanine to serine amino acid (A69S), may be a genetic factor leading to nAMD by affecting the function of the retinal mitochondria [13].

Vascular endothelial growth factor (*VEGF*) is also involved in nAMD development due to formation of angiogenesis and vascular permeability that results in fluid leakage across the blood vessels [1]. It focuses on inhibiting *VEGF* with an intravitreal injection of anti-VEGF agents such as bevacizumab [14] and ranibizumab [15]. While the anti-VEGF therapy was proved to effectively slow the progress of choroidal neovascularisation (CNV), heterogeneity was observed among patients in terms of degree of response and duration of treatment [16], and it was hypothesised that genetic biomarkers that are strongly associated with nAMD development such as the variants of *CFH*, *VEGFA*, *ARMS2* and *HTRA1* genes might be involved in this heterogeneous response [17–20].

Furthermore, pharmacogenetic studies have been increasing to further investigate the varied response to ranibizumab anti-VEGF therapy, especially between high-risk polymorphisms associated with nAMD such as the rs11200638 polymorphism in *HTRA1* gene [6, 21] and rs10490924 polymorphism in *ARMS2* gene [7]. Studies have reported the risk allele of the *HTRA1* gene could lead to overexpression of *HTRA1* protein and possibly affecting the integrity of Bruch’s membrane and stimulate the development of CNV [9]. The inhibition of TGF-β by *HTRA1* could also influence the response to anti-VEGF inhibitors as TGF-β plays an important role in angiogenesis [22]. Regarding the expression of *ARMS2* in the mitochondrial retina, evidence has suggested that variants of the *ARMS2* gene could lead to RPE dysfunction due to mitochondrial DNA damage that accumulates in the retina and RPE. Furthermore, the mitochondria are known to be a major source of superoxide anion in the cell, indicating damages to the mitochondria leads to oxidative stress in nAMD which possibly could affect the response to anti-VEGF inhibitors [22].

However, the current research is inconclusive as there are conflicting findings on the association of rs11200638 and rs10490924 gene polymorphisms in response to ranibizumab therapy [16, 23]. This has led us to evaluate the genetic association of rs11200638 polymorphism in the *HTRA1* gene and rs10490924 polymorphism in the *ARMS2* gene and its responses to ranibizumab therapy among nAMD subjects in Malaysia. We also investigated the correlation between *HTRA1* and *ARMS2* mRNA levels among the subjects responding and not responding to ranibizumab therapy.

## Results

### Sample characteristics

The subjects’ socio-demographic characteristics are presented in Table 1. From 145 nAMD subjects and 145 control subjects, nAMD subjects recorded a significantly higher age (69.10 ± 7.51 years) compared to controls (64.96 ± 10.12 years, *P* < 0.001). Male subjects were recorded with significantly greater frequency (*P* = 0.019) in both nAMD (59.3%) and controls (72.4%) compared to female subjects in both groups.

**Table 1:** Socio-demographic characteristic of subjects

Parameter		nAMD (*n* = 145)	Controls (*n* = 145)	*P* value^a^
Age		69.10 ± 7.51	64.96 ± 10.12	< 0.001^b^*
Gender	Male	86 (59.3)	105 (72.4)	
	Female	59 (40.7)	40 (27.6)	0.019*
Race	Malay	51 (35.2)	60 (41.4)	
	Chinese	88 (60.7)	58 (40.0)	
	Indian	6 (4.1)	27 (18.6)	< 0.001*
Co-morbidities	DM	47 (32.4)	57 (39.3)	0.221
	HPT	90 (62.1)	64 (44.1)	0.002^*^
	DM + HPT	40 (27.6)	36 (24.8)	0.593
Smoking		50 (34.5)	24 (16.6)	< 0.001*

Data are presented as percentages in parentheses

*DM* diabetes mellitus, *HPT* hypertension

^*^Significant *P* value, *P* < 0.05

^a^Chi-square test

^b^Student’s *t* test

A comparison between ethnicities revealed that Chinese subjects were highest in the nAMD group (60.7%); however, among the controls, Malay subjects were reported highest (41.4%). The co-morbidities that reported a significant difference between nAMD and control was hypertension (*P* = 0.002) whereby nAMD had a higher frequency of hypertensive subjects (62.1%) compared to controls (44.1%). Smoking was also observed to be significantly associated with nAMD compared to controls (*P* < 0.001).

### Genotypic and allelic association among nAMD and control subjects

**Fig. 1:**
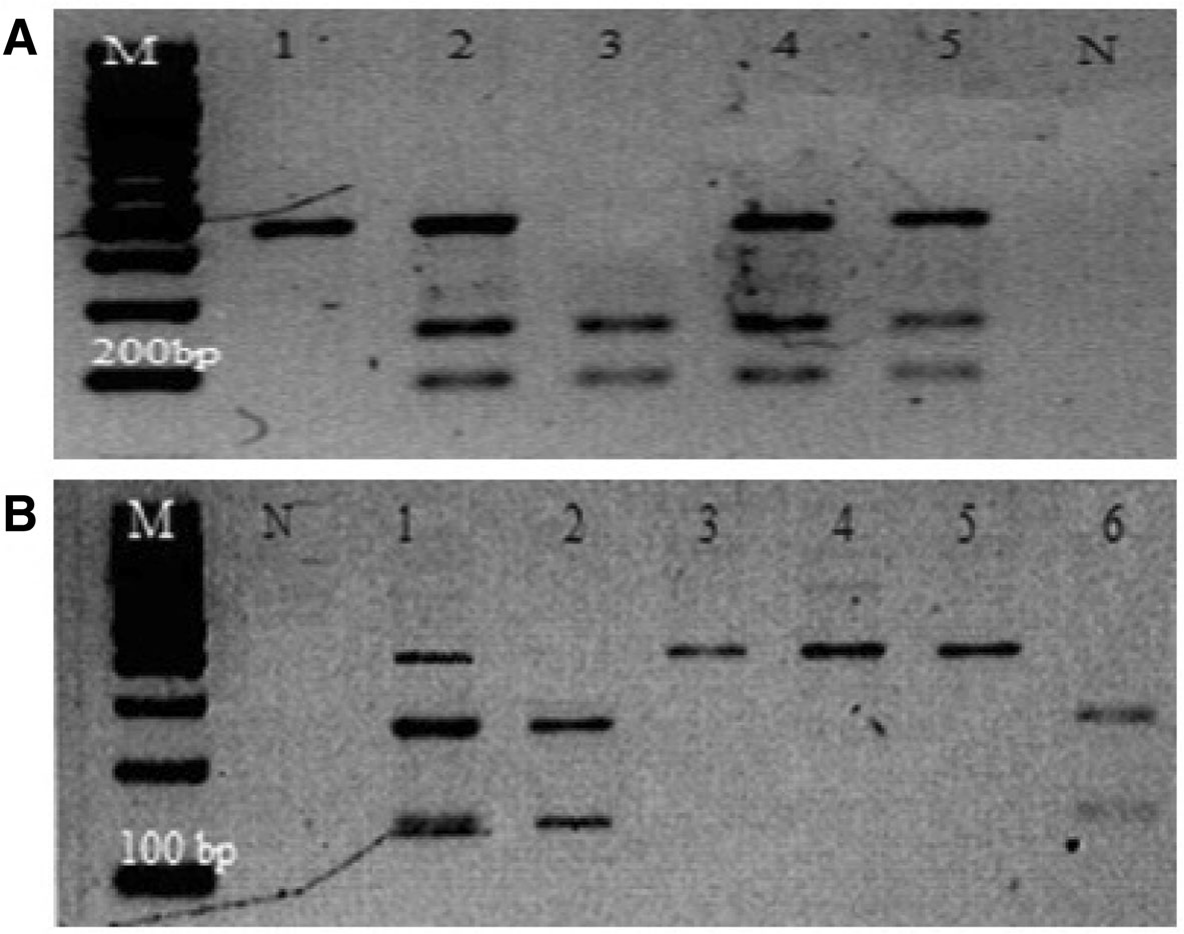
PCR and RFLP product for *ARMS2* rs10490924 (**a**) and *HTRA1* rs11200638 (**b**) gene polymorphisms on 2% agarose gel. RFLP product of *ARMS2* rs10490924 (**a**): Lane 1 shows homozygous GG, 259 bp and 190 bp; lane 2 and lane 6 show heterozygous GT, 449 bp, 259 bp and 190 bp; lane 3 until lane 5 show homozygous TT, 449 bp. RFLP product of *HTRA1* rs11200638 (**b**): lane 1, lane 4, lane 5 and lane 6 show homozygous AA, 385 bp; lane 2 shows heterozygous GA, 385 bp, 246 bp and 139 bp; lane 7 shows homozygous GG, 246 bp and 139 bp

Genotyping was performed for both *ARMS2* rs10490924 and *HTRA1* rs11200638 gene polymorphisms (Fig. 1a, b) and compared between nAMD and control subjects. Genotyping data showed that allelic frequencies were in Hardy-Weinberg equilibrium (*P* > 0.05) except for the nAMD group in *HTRA1* rs11200638 (*P* < 0.05). Both gene polymorphisms showed a significant difference for genotypes and alleles when compared between the two subject groups with a recorded OR = 2.44, CI = 1.75–3.42 (*P* < 0.001) for the *ARMS2* rs10490924 polymorphism and OR = 1.52, CI = 1.07–2.15 (*P* = 0.018) for the *HTRA1* rs11200638 polymorphism (Table 2). A strong linkage disequilibrium is recorded between the two polymorphisms (*D*’ = 0.92, *r*^2^ = 0.68), suggesting the nearly identical association test and odd ratios.

**Table 2:** Genotype association between subjects

Gene variant	W/M	Subjects	WW	WM	MM	*P* value^a^	W (%)	M (%)	*P* value^a^	HWE^b^		OR (95% CI)
										*χ* ^2^	*P*	
*ARMS2* rs10490924	G/T	nAMD	21 (14.5)	57 (39.3)	67 (46.2)		34.1	65.9		2.293	0.318	2.44
		Controls	46 (31.7)	70 (48.3)	29 (20.0)	< 0.001*	55.9	44.1	< 0.001*	0.068	0.966	(1.75–3.42)
*HTRA1* rs11200638	G/A	nAMD	19 (13.1)	47 (32.4)	79 (54.5)		29.3	70.7		6.823	0.033	1.52
		Controls	15 (10.3)	82 (56.6)	48 (33.1)	< 0.001*	38.6	61.4	0.018*	5.386	0.068	(1.07–2.15)

Data are presented as percentages in parentheses

*M* risk allele, *W* non-risk allele, *OR* odds ratio, *CI* confidence interval, *HWE* Hardy-Weinberg equilibrium

^*^Significant *P* value, *P* < 0.05

^a^Chi-square test

^b^*χ*^2^ goodness-of-fit test

Further genetic association analysis was performed between the genotypes of both gene polymorphism with BCVA and CRT of nAMD patients at baseline and 3 and 6 months post-treatment with intravitreal ranibizumab therapy (Table 3). Both homozygous risk genotypes of *ARMS2* rs10490924 and *HTRA1* rs11200638 showed a significantly worse mean BCVA and mean CRT at 6 months post-treatment with mean BCVA 0.62 ± 0.28 logMAR (*P* = 0.046) and 0.61 ± 0.29 logMAR (*P* = 0.044), respectively, and mean CRT 400.1 ± 127.90 μM (*P* < 0.001) and 387.5 ± 129.89 μM (*P* = 0.008), respectively. These findings were supported by the mean changes of BCVA and CRT from the baseline, which also reported significantly worse changes (*P* < 0.05) at 6 months post-treatment for both homozygous risk genotypes in both polymorphisms. In addition, male nAMD subjects were significantly frequent carrying the risk genotype compared to females for both *ARMS2* rs10490924 (56.7%, *P* = 0.032) and *HTRA1* rs11200638 (54.4%, *P* = 0.001).

**Table 3:** Visual acuity and central retinal thickness outcome by genotype among nAMD subjects

		*ARMS2* rs10490924 (*n* = 145)				*HTRA1* rs11200638 (*n* = 145)			
		GG	GT	TT	*P*	GG	GA	AA	*P*
Total (*n*)		21 (14.5)	57 (39.3)	67 (46.2)		19 (13.1)	47 (32.4)	79 (54.5)	
Mean age (years)^a^		66.47 ± 7.31	70.35 ± 7.74	68.87 ± 7.25	0.122^c^	65.95 ± 7.46	69.87 ± 7.79	69.41 ± 7.26	0.137^c^
Gender	Male	8 (38.1)	40 (70.2)	38 (56.7)		6 (31.6)	37 (78.7)	43 (54.4)	
	Female	13 (61.9)	17 (29.8)	29 (43.3)	0.032^c*^	13 (68.4)	10 (21.3)	36 (45.6)	0.001^c*^
Mean BCVA (logMAR) ^a^	Baseline	0.59 ± 0.28	0.56 ± 0.28	0.56 ± 0.25	0.861^b^	0.59 ± 0.27	0.62 ± 0.28	0.53 ± 0.25	0.190^b^
	3 months	0.58 ± 0.28	0.58 ± 0.26	0.61 ± 0.27	0.771^b^	0.58 ± 0.29	0.61 ± 0.27	0.59 ± 0.26	0.906^b^
	6 months	0.45 ± 0.29	0.58 ± 0.28	0.62 ± 0.28	0.046^b^*	0.44 ± 0.31	0.58 ± 0.27	0.61 ± 0.29	0.044^b^*
Mean CRT (μM) ^a^	Baseline	417.8 ± 129.82	388.7 ± 143.95	369.5 ± 134.71	0.357^c^	431.5 ± 128.46	393.9 ± 151.47	366.7 ± 129.63	0.153^c^
	3 months	343.9 ± 165.37	355.3 ± 131.49	353.6 ± 93.17	0.411^b^	350.3 ± 172.62	357.9 ± 128.46	350.5 ± 100.83	0.665^b^
	6 months	323.6 ± 144.39	339.0 ± 129.48	400.1 ± 127.90	< 0.001^b^*	329.2 ± 150.19	341.7 ± 130.29	387.5 ± 129.89	0.008^b^*
Changes in BCVA (logMAR) ^a^	3 months	− 0.01 ± 0.21	0.02 ± 0.24	0.05 ± 0.19	0.263^b^	− 0.003 ± 0.19	− 0.01 ± 0.24	0.06 ± 0.21	0.104^b^
	6 months	− 0.14 ± 0.27	0.03 ± 0.28	0.06 ± 0.29	0.011^b^*	− 0.15 ± 0.26	− 0.03 ± 0.27	0.08 ± 0.29	0.002^b^*
Changes in CRT (μM) ^a^	3 months	− 73.9 ± 98.67	− 33.4 ± 126.33	− 15.8 ± 140.46	0.507^c^	− 81.2 ± 101.09	− 36.1 ± 113.17	− 16.1 ± 143.46	0.141^c^
	6 months	− 94.2 ± 82.17	− 49.7 ± 145.18	30.6 ± 184.27	0.022^c^*	− 102.4 ± 82.16	− 52.3 ± 145.09	20.8 ± 178.85	0.003^c^*

Data are presented as percentages in parentheses

*CRT* central retinal thickness, *BCVA* best-corrected visual acuity

^*^Significant *P* value, *P* < 0.05

^a^Data presented as mean ± standard deviation (SD)

^b^Kruskal-Wallis test

^c^Mann-Whitney *U* test

### Comparison of response to treatment

The nAMD subjects were further classified into responders and non-responders to the intravitreal ranibizumab therapy based on the BCVA and CRT recorded after treatment. Overall, responders recorded a worse mean BCVA (0.66 ± 0.27 logMAR, *P* = 0.001) and worse mean CRT (480.3 ± 153.78 μM, *P* < 0.001) at baseline compared to non-responders. However, at 6 months post-treatment, non-responders recorded a worse mean BCVA (0.67 ± 0.28 logMAR, *P* < 0.001) and worse mean CRT (390.8 ± 145.79 μM, *P* = 0.001) compared to responders. The mean changes in both BCVA and CRT at 3 and 6 months post-treatment from baseline among responders and non-responders were also significantly different (*P* < 0.05). No significant differences (*P* > 0.05) were observed between age and smoking status between the two groups (Table 4).

**Table 4:** Visual acuity and central retinal thickness outcome by response to ranibizumab among nAMD subjects

		Responder (*n* = 54)	Non-responder (*n* = 91)	*P* ^b^
Age		68.33 ± 6.47	69.56 ± 8.07	0.343
Smoking		15 (27.8)	35 (38.5)	0.191
Mean BCVA (logMAR)^a^	Baseline	0.66 ± 0.27	0.51 ± 0.24	0.001*
	3 months	0.57 ± 0.29	0.61 ± 0.24	0.304
	6 months	0.43 ± 0.24	0.67 ± 0.28	< 0.001*
Mean CRT (μM)^a^	Baseline	480.3 ± 153.78	326.9 ± 87.12	< 0.001^c^*
	3 months	348.0 ± 114.79	355.8 ± 124.27	0.642
	6 months	321.5 ± 98.73	390.8 ± 145.79	0.001*
Changes in BCVA (logMAR)^a^	3 months	− 0.09 ± 0.20	0.10 ± 0.19	< 0.001*
	6 months	− 0.23 ± 0.22	0.16 ± 0.23	< 0.001*
Changes in CRT (μM)^a^	3 months	− 132.3 ± 133.05	28.92 ± 83.39	< 0.001^c^*
	6 months	− 158.9 ± 132.02	63.96 ± 119.60	< 0.001^c^*

Data are presented as percentages in parentheses

*CRT* central retinal thickness, *BCVA* best-corrected visual acuity

^*^Significant *P* value, *P* < 0.05

^a^Data presented as mean ± standard deviation (SD)

^b^Mann-Whitney *U* test

^C^Student’s *t* test

When the treatment response group was further analysed based on the genotypes for *ARMS2* rs10490924, a significant difference was observed among the non-responder group in mean changes of CRT at 6 months post-treatment with a highest increase of 97.3 ± 128.36 μM among non-responders carrying the risk genotype (*P* = 0.009). For *HTRA1* rs11200638, the mean BCVA at 3 months post-treatment recorded a significantly worse BCVA among non-responders which carried the non-risk genotype (0.90 ± 0.20 logMAR, *P* = 0.034). A significant association was also observed in a regression analysis between the two response groups and genotypes for each polymorphism with the *ARMS2* rs10490924 using a co-dominant model (*P* < 0.001) and *HTRA1* rs11200638 using a recessive model (*P* < 0.001) (Table 5).

**Table 5:** Visual acuity and central retinal thickness outcome by response to ranibizumab and genotypes

		Responders (*n* = 54)				Non-responders (*n* = 91)			
*ARMS2* rs10490924		GG (*n* = 16)	GT (*n* = 22)	TT (*n* = 16)	*P* ^c^	GG (*n* = 5)	GT (*n* = 35)	TT (*n* = 51)	*P* ^c^
Age		66.68 ± 7.88	68.77 ± 6.97	69.38 ± 3.70	0.469	65.80 ± 5.81	71.34 ± 8.12	68.71 ± 8.07	0.187
Smoking		5 (31.3)	8 (36.4)	2 (12.5)	0.251	2 (40.0)	16 (45.7)	17 (33.3)	0.509
Mean BCVA (logMAR)^a^	Baseline	0.58 ± 0.27	0.67 ± 0.27	0.72 ± 0.28	0.328^b^	0.64 ± 0.35	0.49 ± 0.26	0.51 ± 0.22	0.509^b^
	3 months	0.51 ± 0.25	0.54 ± 0.27	0.67 ± 0.36	0.253	0.82 ± 0.26	0.61 ± 0.25	0.59 ± 0.23	0.191^b^
	6 months	0.33 ± 0.19	0.48 ± 0.26	0.45 ± 0.22	0.119^b^	0.82 ± 0.26	0.65 ± 0.28	0.67 ± 0.28	0.420^b^
Mean CRT (μM)^a^	Baseline	432.7 ± 107.73	490.5 ± 159.19	514.1 ± 180.79	0.307	370.2 ± 192.43	324.7 ± 86.96	324.1 ± 73.19	0.525
	3 months	327.1 ± 88.53	367.3 ± 138.55	342.5 ± 103.94	0.562	397.8 ± 320.3	347.8 ± 128.34	357.1 ± 90.37	0.406^b^
	6 months	307.7 ± 85.37	323.6 ± 103.78	332.3 ± 108.36	0.78	374.4 ± 269.59	348.7 ± 143.92	421.4 ± 127.03	0.072
Changes in BCVA (logMAR)^a^	3 months	− 0.07 ± 0.13	− 0.13 ± 0.22	− 0.05 ± 0.23	0.462^b^	0.18 ± 0.30	0.12 ± 0.21	0.08 ± 0.17	0.651^b^
	6 months	− 0.24 ± 0.16	− 0.19 ± 0.24	− 0.26 ± 0.23	0.593	0.18 ± 0.30	0.16 ± 0.21	0.15 ± 0.24	0.960^b^
Changes in CRT (μM)^a^	3 months	− 105.6 ± 59.04	− 123.2 ± 122.81	− 171.6 ± 188.21	0.349	27.6 ± 136.82	23.1 ± 92.01	33.0 ± 72.25	0.865
	6 months	− 125.0 ± 52.98	− 166.8 ± 135.36	− 181.8 ± 176.76	0.454	4.2 ± 85.65	23.9 ± 94.53	97.3 ± 128.36	0.009*
Logistic regression^d^	Co-dominant ^e^		*P* < 0.001^*^, OR = 3.15, 95% CI (1.75–5.67)						
*HTRA1* rs11200638		GG (*n* = 15)	GA (*n* = 21)	AA (*n* = 18)		GG (*n* = 4)	GA (*n* = 26)	AA (*n* = 61)	
Age		66.53 ± 8.13	68.95 ± 7.07	69.11 ± 3.66	0.455	63.75 ± 4.11	70.62 ± 8.39	69.49 ± 8.04	0.286
Smoking		4 (26.7)	9 (42.9)	2 (11.1)	0.087	2 (50.0)	13 (50.0)	20 (32.8)	0.284
Mean BCVA (logMAR)^a^	Baseline	0.55 ± 0.25	0.69 ± 0.28	0.69 ± 0.27	0.192^b^	0.73 ± 0.34	0.55 ± 0.26	0.48 ± 0.22	0.180^b^
	3 months	0.49 ± 0.25	0.58 ± 0.29	0.62 ± 0.33	0.521	0.90 ± 0.20	0.63 ± 0.25	0.58 ± 0.23	0.034^b^*
	6 months	0.32 ± 0.19	0.48 ± 0.26	0.46 ± 0.22	0.059^b^	0.90 ± 0.20	0.67 ± 0.25	0.65 ± 0.29	0.191^b^
Mean CRT (μM)^a^	Baseline	444.7 ± 99.74	487.1 ± 168.15	502.1 ± 174.90	0.556	382.0 ± 220.09	318.8 ± 80.19	326.7 ± 77.79	0.406
	3 months	332.4 ± 88.99	380.5 ± 141.31	323.2 ± 94.33	0.251	417.5 ± 366.34	339.7 ± 116.68	358.6 ± 102.01	0.519^b^
	6 months	313.4 ± 85.14	333.1 ± 109.14	314.6 ± 100.66	0.794	388.3 ± 309.23	348.7 ± 146.94	408.9 ± 130.38	0.212
Changes in BCVA (logMAR)^a^	3 months	− 0.05 ± 0.10	− 0.12 ± 0.23	− 0.08 ± 0.23	0.735^b^	0.18 ± 0.36	0.08 ± 0.20	0.10 ± 0.18	0.870^b^
	6 months	− 0.23 ± 0.16	− 0.22 ± 0.25	− 0.24 ± 0.22	0.945	0.18 ± 0.35	0.12 ± 0.18	0.17 ± 0.24	0.664^b^
Changes in CRT (μM)^a^	3 months	− 112.3 ± 54.30	− 106.6 ± 115.37	− 178.9 ± 183.41	0.191	35.5 ± 156.67	20.9 ± 73.19	31.9 ± 83.25	0.846
	6 months	− 131.3 ± 48.17	− 154.0 ± 135.01	− 187.5 ± 171.50	0.474	6.3 ± 98.75	29.9 ± 91.8	82.3 ± 128.03	0.107
Logistic regression^d^	Recessive^e^		*P* < 0.001^*^, OR = 4.42, 95% CI (2.11–9.25)						

*CRT* central retinal thickness, *BCVA* best-corrected visual acuity, *OR* odds ratio, *CI* confidence interval

^*^Significant *P* value, *P* < 0.05

^a^Data presented as mean ± standard deviation (SD)

^b^Kruskal-Wallis test

^c^One-way ANOVA

^d^Adjusted for age and gender

^e^Regression model with the least *P* value

### Gene expression analysis

**Fig. 2:**
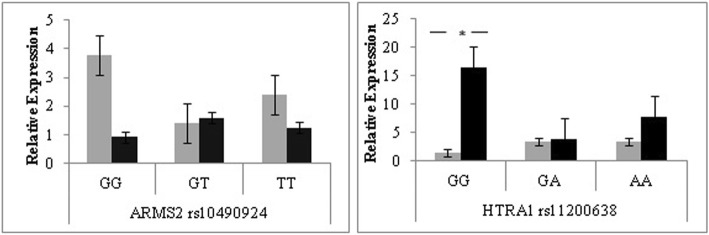
Relative expression levels of *ARMS2* and *HTRA1* gene variant among responders (*n* = 20) and non-responders (*n* = 20) determined by the ∆∆Ct calculation. **a** mRNA levels according to *ARMS2* rs10490924 genotype. **b** mRNA levels according to *HTRA1* rs11200638 genotypes. A significant difference was only observed between responder and non-responder groups for homozygous non-risk GG genotype of *HTRA1* rs11200638 (*P* = 0.032). Bars, relative expression ± standard error mean. Light bars, responder group. Dark bars, non-responder group

The levels of mRNA for the *HTRA1* gene showed a significant difference between non-responders and responders for the homozygous non-risk GG genotype with the responders having a 15-fold higher expression of the *HTRA1* gene compared to non-responders (*P* = 0.032). No significant difference was observed for the *ARMS2* expression levels between the two response groups (Fig. 2).

## Discussion

Over the years, an increasing number of biomarkers related to the development of nAMD have been discovered, and the most frequent findings are polymorphisms of the *ARMS2* and *HTRA1* genes. The heterogeneity in response to anti-VEGF treatment among nAMD patients has resulted in increasing pharmacogenetic studies on the association of possible high-risk biomarkers related to nAMD which respond to anti-VEGF treatment. Previously, we have investigated the pharmacogenetic response of *CFH Y402H* polymorphism with ranibizumab therapy but reported no significant association among Malaysian subjects [24].

Aetiology of nAMD is known as a multifactorial disease caused by environmental and genetic factors [3]. Increasing age is known as the strongest risk factor associated with AMD [1, 3], followed by smoking [2]; this could also be why male gender is significant in the present study as most smokers are among the male subjects. Gender risk is inconsistent; there are studies reporting females at higher risk of AMD [3, 25]; however, there are also studies reporting males at higher risk [26, 27]. The association of Chinese ethnicity in the present study is similar to the findings from Singapore, which also has a multi-ethnic population, that AMD was more common among the Chinese and Indian compared to Malays [27]. A recent finding from Malaysia also reported the Chinese had a significantly higher frequency of receiving ranibizumab treatment compared to Indians and Malays [28]. Furthermore, the Age-Related Eye Disease Study Research Group [29] and Vassilev et al. [30] similarly report an association between diabetes and hypertension with a risk of developing nAMD.

Despite the environmental factors, genetic factors, such as genetic polymorphisms of *ARMS2* and *HTRA1* genes, are commonly studied in relation to nAMD with significant findings in various populations, such as findings from northern China [19], India [31], Han Chinese [8], Israel [6] and Poland [32]. We also reported an association of *complement factor H* gene [24, 33], *VEGF + 405 G/C* [34] and *complement factor 3 R102G* [35] gene polymorphisms with AMD among Malaysians. A recent pharmacogenetic study on *CFH* rs1061170 among Malaysians reported no significant association with response to ranibizumab therapy [24]. In the present study, a significant association of *ARMS2* rs10490924 and *HTRA1* rs11200638 gene polymorphisms was reported among the nAMD patients. Since both *ARMS2* rs10490924 and *HTRA1* rs11200638 gene polymorphisms have increasing reports of being associated with nAMD susceptibility, this led us to further investigate the relation of these polymorphisms with the heterogeneity response of nAMD patients to the ranibizumab treatment.

In this study, the response to ranibizumab was measured with regard to both anatomical (CRT) and functional (BCVA) aspects in correlation with *ARMS2* rs10490924 and *HTRA1* rs11200638 gene polymorphisms. To further evaluate the variable response to ranibizumab, the nAMD subjects were categorised into responders and non-responders based on the BCVA and CRT outcomes. There were also previous studies that measure the treatment outcome by categorising the subjects by their response to treatment; however, there was no uniformity and it could be one of the reasons for the conflicting findings on nAMD pharmacogenetic studies [36].

A significant association was reported after a logistic regression analysis in the present study between the two treatment response groups and the *ARMS2* rs10490924 and *HTRA1* rs11200638 polymorphisms, suggesting that the variable response to treatment might be affected by changes in the 10q26 chromosome biomarkers [7, 23]. These findings are further supported by the previous studies which reported that the risk allele *ARMS2* rs10490924 and *HTRA1* rs11200638 polymorphism was associated with a poor response to ranibizumab based on changes in BCVA [21, 37–39] and CRT [40]. However, the distribution of the *HTRA1* rs11200638 alleles in the present study were not consistent with HWE among the nAMD groups which reasons could be due to *HTRA1* rs11200638 as being strongly associated with risk of nAMD among Asian populations [17]. The nAMD group in the present study was comprised of Malaysian subjects only and might explain the deviation from HWE.

Higher mean changes in CRT was observed at 6 months post-treatment among non-responders with homozygous risk *ARMS2* rs10490924 TT genotype (*P* = 0.009) compared to non-responders with the normal genotype that have a smaller mean change in CRT. This suggests that subjects carrying the homozygous risk *ARMS2* rs10490924 TT genotype are more likely to not respond to ranibizumab treatment compared to those with normal genotypes. These findings are consistent with previous studies such as those in Korea [17], Australia [39] and Poland [32]. However, there are also contradictory findings whereby the association of *ARMS2* rs10490924 with ranibizumab response was not observed [7, 23], even in major studies, such as the IVAN study [41] and CATT trial [42]. The underlying discrepancies in this contradictory finding could be related to several aspects such as the differences in the treatment regimen of the patients. The present study only included patients receiving a treat and extend regimen of ranibizumab whereas the IVAN and CATT study included both monthly and pro re nata regimen treatment groups in their pharmacogenetic analysis. Second, the ethnic differences could result in the different associations as the present study included only Malaysians and findings from a Population Architecture using Genomics and Epidemiology study reported that risk associations of *CFH* rs1061170 and *ARMS2* rs10490924 in Europeans did not generalise to the non-European populations [43]. Findings from a large study conducted in India reported variants that were associated with AMD in the Western populations; the variants of *CFH*, *C2* and *complement factor B* were contradictory with findings in India. Furthermore, the variants in *ARMS2* and *HTRA1* genes were found to be associated with increased risk of AMD in the INDEYE study, but not in the Western populations, which further suggests the effect of ethnic differences in AMD polymorphism studies [31].

Similarly, the non-responder group between *HTRA1* rs11200638 genotypes also showed a significant difference, but only on mean BCVA at 3 months post-treatment with normal GG genotype (0.90 ± 0.20 logMAR) having the worse mean BCVA compared to risk AA genotype (0.58 ± 0.23 logMAR, *P* = 0.034). This contradicts the *ARMS2* rs10490924 findings, as the non-responders with the worst visual acuity were among the subjects carrying the risk genotype of *ARMS2* rs10490924. This could be because the mean baseline BCVA was initially worse among subjects with *HTRA1* rs11200638 GG genotypes. However, the risk AA genotypes reported a higher loss of BCVA from the baseline compared to the normal GG genotype which did not lose further vision at 6 months post-treatment.

It is suggested that the response to treatment could also be affected by the baseline vision, due to a phenomenon known as the ceiling effect, whereby subjects with a worse baseline BCVA were less likely to lose further vision and subjects with better BCVA were less likely to improve [44, 45]. This has also been observed in large studies, including the MARINA [46] and ANCHOR [47] studies which reported that the baseline BCVA is a strong predictor of treatment response. The poor baseline BCVA was also reported to be related with sub-retinal fluid thickness at baseline and these subjects had a greater possibility of gaining vision compared with subjects that had a better BCVA at baseline [48]. The UK Age-Related Macular Degeneration EMR Users Group was also in accordance with the present findings as they reported that those with a better baseline VA had a mean drop of six letters lost; however, those with a worse baseline VA only had a mean loss of three letters by 52 weeks post-treatment [49].

Due to the strong linkage disequilibrium among the *ARMS2* and *HTRA1* genes, it remains unclear which variants are at higher risk of nAMD pathogenesis; thus, the correlation of *ARMS2* and *HTRA1* variants to ranibizumab treatment response remains unclear. However, studies are suggesting that the risk allele might lead to changes in transcriptional activity and, thus, affect the proteins normal activity resulting in a differing response to the ranibizumab treatment [21].

In the present study, since we observed significant associations between *ARMS2* rs10490924 and *HTRA1* rs11200638 polymorphisms in response to ranibizumab and the strong linkage disequilibrium among the two variants, we further investigated the mRNA levels of both variants among the two treatment response groups. A significant association was only observed in the mRNA expression of *HTRA1* rs11200638 GG genotype with the non-responders having the highest *HTRA1* expression compared to the responders. The high expression of *HTRA1* GG genotype was also reported by Yang et al. [10] and DeWan et al. [9] in a study between AMD and healthy controls whereby the AMD subjects had a higher expression of *HTRA1*. The overexpression of *HTRA1* has been related to the alteration of Bruch’s membrane, resulting in the invasion of the extracellular matrix with choroid capillaries and inhibition of the angiogenesis and extracellular matrix deposition regulator (*TGF-β*) [10]. Thus, the worse BCVA and high thickness of CRT among the GG genotypes of non-responders in the present study could be related to the overexpression of *HTRA1* genes resulting to the poor response to the ranibizumab treatment.

The present study focused on a targeted SNP analysis of *ARMS2* and *HTRA1* genes due to the high risk of rs10490924 and rs11200638 polymorphisms with development of nAMD and their significant association with response to ranibizumab therapy such as reported in China [19], Korea [50] and Spain [7]. The SNPs were selected based on its functional significance to the heterogeneous response to ranibizumab, the location of the SNPs and a minor allele frequency of more than 10% as suggested by Pettersson et al. [51] and Tabangin et al. [52] in the selection of SNPs for genetic association studies.

### Study limitations

Despite the findings reported in this study, there are limitations that should be taken into consideration. The study design itself is a limitation as this is an unmatched comparative case-control study. A matched case-control study is advantageous in controlling cofounders that could alter the exposure and outcome relationship in an analysis. However, the present study with an unmatched case-control could better examine the impact of the cofounders and further provide evidence on significant risk factors related to nAMD. The lack of CNV subtypes is one of the major limitations as performing analysis based on the CNV subtypes (predominantly classic CNV, minimally classic CNV and occult CNV) could provide more specific information on which CNV is related with the different response to ranibizumab. A short follow-up period of 6 months post-treatment is also a limitation due to time constraint in the present study. Although the small sample size is a significant sample size for a two-centre study in a Malaysian population, future studies could be conducted in a larger sample size in multiple centres around Malaysia to confirm the pharmacogenetic findings. Furthermore, the variable criteria in grouping the responders/good response and non-responders/poor response to the ranibizumab treatment could also be one of the factors affecting the pharmacogenetic results, as there are contradictory findings in previous studies. The present study only focused on two variants of *ARMS2* and *HTRA1* genes which do not exclude the possibility of other variants in the two genes or in other high-risk genes (*CFH*, *VEGFA* and *C3*) or even a combined effect of the polymorphisms that might play a role in the heterogenous response to ranibizumab therapy. Future studies could also sequence other rare variants in the high-risk genes and this might provide a more depth information on pharmacogenetic associations of nAMD.

## Conclusions

A significant association was observed between *ARMS2* rs10490924 and *HTRA1* rs11200638 polymorphisms in response to the ranibizumab treatment among nAMD subjects. An overexpression of mRNA in the *HTRA1* GG genotype was also reported and could contribute to the non-responders’ reaction to ranibizumab. Despite the reported data, future studies are needed to confirm the findings with a larger sample size and more stringent criteria which accounts for the limitations of the present study.

## Materials and methods

This study was approved by Universiti Putra Malaysia (UPM), Universiti Kebangsaan Malaysia (UKM) ethics committee and Medical Research Ethics Committee (MREC), Ministry of Health, Malaysia (MOH). This study was registered with the National Medical Research Registry with the registration number NMRR-14-1176-21475.

### Data collection

A prospective cohort study was conducted from September 2014 to February 2016 in ophthalmology clinics at two tertiary centres, Universiti Kebangsaan Malaysia Medical Centre (UKMMC) and Hospital Selayang. A total of 145 nAMD subjects were selected as case subjects from a total of 158 nAMD subjects, as the remaining 13 subjects failed at follow-up. Informed consent was obtained from all the subjects prior to starting the study.

### Patient’s criteria

All subjects underwent comprehensive ophthalmologic examinations, including BCVA test by Snellen chart, fluorescein angiography (FA) and central retinal thickness analysis using optical coherence tomography (OCT). The subjects were then diagnosed as nAMD by ophthalmologists at each centre based on the clinical examinations performed. None of the subjects were previously treated for nAMD.

All subjects were aged above 50 years old with confirmed presence of exudation involving macula and a follow-up appointment of 6 months after the first injection of ranibizumab (Lucentis; Novartis Pharma AG, Basel, Switzerland and Genentech Inc., South San Francisco, California, USA). They were eligible for this study. Subjects who do not fulfil the mentioned inclusion criteria and had a CNV secondary to other causes, such as inflammatory diseases, trauma, hereditary diseases, pathologic myopia, angioid streaks, other retinal diseases other than AMD and eyes previously submitted to posterior vitrectomy were excluded from this study.

### Control’s criteria

Control subjects with no previous history of nAMD, absence of other retinal disorders except mild cataract, no visual impairment and no family history of nAMD were recruited in both centres during routine ophthalmic examination. Subjects with severe cataracts were excluded from control subjects.

### Measures of response to treatment

Socio-demographic factors, medical history, smoking history and the complications of nAMD were retrieved from the medical records of the subjects. Each nAMD subject received 0.5 mg/0.05 ml ranibizumab per intravitreal injection following the treat and extend regimen and was followed up for 6 months. The BCVA and central retinal thickness (CRT) at baseline and 6 months were compared and evaluated with regard to the treatment. Those with an improvement of three lines or greater in the Snellen chart and/or a resolution of more than 100 μM decrease in CRT at 6 months post-treatment compared to baseline were regarded as responders, while the non-responders had no improvements.

### Gene and SNP selection

Based on the obtained literature reviews, *HTRA1* rs11200638 and *ARMS2* rs10490924 gene polymorphisms were among the significantly associated variants with response to ranibizumab therapy in nAMD and, thus, were selected as a candidate SNP in the present study. Furthermore, the SNP was selected based on the functional significance, location and minor allele frequency of more than 10%. *HTRA1* rs11200638 and *ARMS2* rs10490924 have a minor allele frequency of 0.241 and 0.255, respectively, as reported by the National Center of Biotechnology Information (NCBI).

### DNA extraction and genotyping

Peripheral blood was collected from all the subjects and the genomic DNA was extracted using a QIAamp DNA blood mini kit (Qiagen, Valencia, California, USA) and stored at − 20 °C for further analysis. The genotyping of the *HTRA1* rs11200638 and *ARMS2* rs10490924 gene polymorphisms was performed using the polymerase chain reaction-restriction fragment length polymorphism (PCR-RFLP) method. The PCR amplification was performed in a thermocycler (Thermo Fisher Scientific, Waltham, MA, USA) with a total volume of 25 μl for each polymorphism, including the following primers: forward: 5′-ATGCCACCCACAACAACTTT-3′ and reverse: 5′-CGCGTCCTTCAAACTAATGG-3′ for *HTRA1* rs11200638 polymorphism and forward: 5′-TACCCAGGACCGATGGTAAC-3′ and reverse: 5′- GAGGAAGGCTGAATTGCCTA-3′ for *ARMS2* rs10490924 polymorphism. Genomic DNA amplification was performed for 35 cycles of denaturation at 95 °C for 30 s, annealing at 57 °C (*HTRA1* rs11200638), 52 °C (*ARMS2* rs10490924) for 30 s and extension at 72 °C for 45 s, followed by the final extension at 72 °C for 5 min.

The amplified product of *HTRA1* rs11200638 (385-bp fragment) and *ARMS2* rs10490924 (449-bp fragment) was then digested with the *EagI* (rs11200638) and *PvuII* (rs10490924) restriction enzymes (New England Biolabs, Beverly, MA, USA) at 37 °C for 3 h. The digested products were separated on 2% agarose gels and visualised using the UV alpha imager (Alpha Innotech, San Leandro, California). Random samples were chosen to validate the results, and identical results were obtained.

### RT-PCR and quantitative real-time PCR

From the 145 nAMD subjects recruited, 20 responders and 20 non-responders to ranibizumab treatment were randomly selected to evaluate the mRNA expression of the *HTRA1* and *ARMS2* genes. Total RNA was isolated from whole blood samples using the Tempus Spin RNA Isolation Reagent Kit (Applied Biosystems, California, USA). The cDNA synthesis was then conducted using the QuantiTect Reverse Transcription Kit (Qiagen, Valencia, California, USA). Following the synthesis of cDNA, quantitative real-time PCR was performed with the QuantiNova SYBR Green PCR Kit (Qiagen, Valencia, California, USA) using the MiniOpticon Real-time PCR System (Bio-rad Hungary Ltd) to evaluate the level of gene expression for the *HTRA1* and *ARMS2* genes. The primers for *HTRA1* expression assay were forward: 5′-CGGAAGATGGACTGATCGTGAC-3 and reverse: 5′-GGTGATGGCTTTTCCTTTGGC-3′ and the primers for *ARMS2* expression assay were forward: 5′-GATGGCAAGTCTGTCCTCCT-3′ and reverse: 5′-TTGCTGCAGTGTGGATGATAG-3′. The housekeeping gene, *β-actin*, was used as an endogenous control in the normalisation of each sample. The samples were prepared in triplicate in a final volume of 20 μl with real-time PCR conditions at 95 °C for 3 min followed by 40 cycles of 95 °C for 10 s and 51 °C for 1 min and a florescence measurement. The relative gene expression was determined by a ∆∆Ct calculation.

### Statistical analysis

The logarithm of the minimal angle of resolution (logMAR) was derived from the BCVA recorded in the Snellen eye examination chart for further statistical analysis. The Hardy-Weinberg equilibrium and linkage disequilibrium were verified using Haploview software. Data on normal continuous variables was compared using Student’s *t* test and one-way analysis of variance (ANOVA), and skewed continuous variables were compared using the Mann-Whitney *U* test and Kruskal-Wallis test whereas categorical variables were compared using the chi-square test. Furthermore, a logistic regression analysis was performed to determine the association between genetic variants of *HTRA1* rs11200638 and *ARMS2* rs10490924 to ranibizumab treatment. All statistical analyses were performed using SPSS version 21.0 (SPSS Inc., Chicago, IL, USA), and *P* < 0.05 was considered to be statistically significant.
